# Traumatic Brain Injury: Contemporary Challenges and the Path to Progress

**DOI:** 10.3390/jcm12093283

**Published:** 2023-05-05

**Authors:** John K. Yue, Hansen Deng

**Affiliations:** 1 Department of Neurological Surgery, University of California, San Francisco, CA 94110, USA; 2 Brain and Spinal Injury Center, Zuckerberg San Francisco General Hospital, San Francisco, CA 94110, USA; 3 Department of Neurological Surgery, University of Pittsburgh Medical Center, Pittsburgh, PA 15261, USA; dengh3@upmc.edu

Traumatic brain injury (TBI) remains a leading cause of death and disability worldwide, and its incidence is increasing. In the United States (US) alone, there were over 2 million emergency department (ED) visits, 220,000 hospitalizations, and 64,000 deaths annually per Centers for Disease Control and Prevention estimates in 2021 [1]. Recent large multicenter studies have shown that an estimated 10% of moderate to severe TBI patients die within 6 months, and an additional 20% are fully dependent in all aspects of care [2]. In mild TBI, 30–56% have not recovered to their functional baseline at 6–12 months post-injury [3,4]. Accepted criteria for clinical diagnosis have been established and commonly consist of external force trauma to the head that causes, at minimum, alteration of consciousness [5]. Biomechanical loading types of closed head injury include impact (direct collision of brain parenchyma with cranial vault, e.g., coup-contrecoup), impulse (inertial forces acting on brain tissue during translational or rotational injury, e.g., axonal shear, deep-seated hematomas, whiplash injuries), and static/quasi-static loading (constant and gradual compressive force, e.g., crush injuries) [6]. Penetrating and blast-type injuries confer additional challenges and management considerations. Presenting symptoms are often heterogeneous in type and severity, and can range from mild post-concussional symptoms to focal neurologic deficits, obtundation, coma, and death. Prompt diagnosis of TBI is paramount for triage to clinical treatment pathways and requires understanding of updated diagnostic methodologies and tools. Over the past decade, significant progress in triage, treatment, diagnosis, and prognosis has improved understanding of the contemporary gaps in care and their solutions.

The importance of expeditious assessment of clinical signs and structural injuries for lifesaving care cannot be overstated. The accepted contemporary framework at major trauma centers consists of the presenting Glasgow Coma Scale (GCS) score for rapid evaluation of potential neurologic compromise, and head computed tomography (CT) scan as the gold standard for localization of traumatic intracranial pathologies [7,8,9]; in combination with laboratory and ancillary clinical data, GCS and CT findings form the basis for TBI diagnosis, severity classification, and triage to the level of care within the current clinical paradigm. Challenges in TBI diagnosis consist of presentation and resource factors. Presenting symptoms can vary widely, and may be confounded by baseline neurologic or mental health conditions, age, medical history and frailty, concomitant medications, and substance use or intoxication. Additionally, challenges to precise TBI severity classification include heterogeneities in the types of traumatic intracranial lesions, coexisting multisystem trauma, and evolving secondary injuries. Integration of a validated framework for neuroimaging assessment of TBI based on modality, lesion type, location, and volume, is fundamental to improving TBI severity classification systems [10,11]. Magnetic resonance imaging (MRI) is more sensitive to small contusions and axonal injuries—which are not readily seen on CT—and has been shown to identify intracranial abnormalities in 27% of CT-negative TBI patients [12]. While certainly not all symptomatic TBI patients require an acute MRI, those who remain persistently symptomatic in a manner incongruous with their clinical presentation or CT findings may be considered for MRI to evaluate for potential CT-occult intracranial pathology.

Blood-based biomarkers with specificity for central nervous system (CNS) injuries have shown promise as diagnostic markers for structural brain injury, as their presence in the circulation is indicative of blood-brain barrier disruption. Robust findings from the prospective, multicenter ALERT-TBI study [13] led to initial clearance of plasma glial fibrillary acidic protein (GFAP) and ubiquitin carboxy-terminal hydrolase-L1 (UCH-L1) to aid in determining the need for head CT within 12 h of TBI by the US Food and Drug Administration (FDA) in 2018. In March 2023, the FDA provided clearance for a clinical assay platform capable of producing GFAP and UCH-L1 results within 18 min, which has primarily been applied to “ruling out” the need for a head CT, given the assay’s negative predictive value of 0.994 for intracranial injury on CT [14]. Expeditious acquisition and processing of blood-based biomarkers propound additional value in settings where neuroimaging is not readily available, or confounding medications and/or substances are on board. Expanded indications for their use, e.g., determining the need to acquire specific neuroimaging sequences and/or utility beyond 12 h post-injury, for GFAP, UCH-L1, and other candidate CNS biomarkers constitute important next steps, as does their inclusion into relevant diagnostic paradigms and prognostic models.

Improved understanding of local and global cerebral physiology after acute TBI has been fundamental to advancing treatment strategies. The 2006 Brain Trauma Foundation guidelines remain the reference of choice for operative indications of cranial trauma [15]. Updated indications and considerations were made in 2019 and 2020 for primary and secondary decompressive craniectomy [16,17]. Refinement of protocols for integrated interpretation and use of multimodal intracranial monitoring data (intracranial pressure, brain tissue oxygenation, cerebral blood flow) [18,19] are ongoing as part of important multicenter randomized clinical trials [20]. Timing and indications for bone flap replacement after decompressive craniectomy remain active areas of investigation [16]. Assessment of local and overall cerebral autoregulation using the cerebrovascular pressure reactivity index and determining dynamic cerebral perfusion pressures have gained wider adoption with the advent of minute-by-minute physiology monitoring and multimodal data capture systems [21], which should be considered in well-resourced, modern neurological intensive care units. Electrocorticography for monitoring of spreading and peri-infarct depolarizations has shown utility in capturing perfusion mismatches and may guide early therapeutic interventions targeting reversible causes of hypotension, metabolic derangements, focal cortical pathology, hyperthermia, and nonconvulsive seizures to reduce secondary injury and improve neurologic outcomes [22,23]. The implementation of high-density physiology monitoring systems has enabled large-scale data collection that harnesses deep analytics and emerging machine learning approaches to better understand and refine the optimal indications for treatment and responses to acute interventions for cerebral and multisystem insults [24,25,26].

While rates of neurosurgical procedural interventions and mortality are low for GCS 13–15 TBI (3–9% and 1–2%, respectively) [27,28], neurologic deterioration can occur acutely in this population and patients with GCS deficits or intracranial neuroimaging findings should receive vigilant clinical monitoring and treatment [29]. Recent studies have bolstered the recognition of non-“mild” TBI symptomatology and sequelae that compromise the return to baseline function. Outcomes and associated deficits are multidimensional, comprising functional, post-concussional, mental health, neurocognitive, sleep, pain, quality of life, and economic self-sufficiency domains, amongst others [30]. Thus, prediction models for outcome should be cognizant of the limitations of dichotomizing outcome measures into “good/poor” or “favorable/unfavorable” to more comprehensively inform the field, given that outcomes are by nature contingent upon injury severity. Post-TBI symptomatology can traverse domains and remain persistent in subsets of patients, particularly when under-recognized or in the presence of maladaptive coping strategies, which often beget further injuries [31]. Hence, validation of candidate risk factors specific to patients with “less severe” brain injuries, in whom demographic and medical history factors often outweigh clinical and radiologic injury variables in predicting outcomes [32], will enable precision medicine approaches to TBI prognostication. These risk factors may play a role in differential trajectories of long-term outcomes, which do not stop at traditional endpoints of 6–12 months. Across the spectrum of TBI severity, multicenter prospective data have shown continued recovery across 1–5 years post-injury [33], establishing TBI as a chronic disease in addition to its status as an injury and event. The impact of polytrauma on TBI management and outcome [34], and corresponding multidisciplinary triage and assessment, constitute important areas of ongoing research.

Accordingly, for TBI patients with more severe initial injuries, prognostication of dichotomized outcomes at 6 months does not capture the totality of TBI outcomes, as subsets of patients continue to recover and improve functionally beyond 2 years [35]. Vigilance in managing expectations and reducing therapeutic nihilism in moderate and severe TBI is increasingly recognized by critical care clinicians [36]. It is important to consider the limitations of validated prognostic tools, such as the International Mission for Prognosis and Analysis of Clinical Trials in TBI (IMPACT) [37] and the Medical Research Council Corticosteroid Randomisation After Significant Head Injury (MRC CRASH) [38] calculators, which include only input variables collected at the time of injury, and do not account for detailed demographic predictors or in-hospital treatment effects. Updated prognostic models should consider the inclusion of dynamic predictors from the emergency and acute treatment course. Older TBI patients, due to higher frailty [39], changes in cerebral anatomy and physiology, and differences in plasma CNS biomarker levels after TBI [40], constitute a distinct population that may require targeted risk stratification and outcome prognostication. Likewise, assessment and prognostication in pediatric TBI require unique considerations due to their bimodal age distribution of injury (0–4 and 15–24 years) [41], guidelines for triage to ionizing radiation [42], and variable symptomatology and sequelae contingent upon developmental age, neurological and mental health history, and caregiver support [43].

Time to diagnosis and availability of diagnostic modalities vary based on setting, for example, between well-resourced urban centers with neurotrauma specialists, neurosurgical and intracranial monitoring capabilities, and rural/remote regions with logistical challenges that rely on interfacility transfers and referrals [44,45]. Formation of responsive regional networks for emergency and acute care neurotrauma consultations and referrals can reduce barriers to access for less-resourced centers. There is differential awareness of the need for TBI prevention, and the multiplicative risks of repetitive TBI on poor outcomes [46], across patients, caregivers, communities, and healthcare settings, due to variability in the understanding of injuries that constitute TBI and when to seek care, as well as expectant challenges and progress during short-term and longitudinal recovery. In developed nations such as the USA, 58% of adult patients with GCS 13–15 TBI reported not receiving TBI-related education from their treating institution, 39% of CT-positive patients reported no outpatient clinical follow-up [47], and 21% reported increased socioeconomic strain at 1 year [48]. Societal, institutional, and governmental resources to systematically reduce health disparities, improve community TBI education, and develop post-acute and outpatient care networks for TBI constitute imperative healthcare gaps and opportunities for care advancement.

The development, implementation, and refinement of best practice data standards, as evidenced by the US National Institute of Neurological Disorders and Stroke TBI Common Data Elements Initiative [49,50], have increased the effectiveness, quality, and reproducibility of neurotrauma studies, potentiated the conception and investigation of research questions directly relevant to patient care, and enabled global data sharing and education of new clinical investigators. Ongoing adoption and refinement of validated data standards will bolster and empower advancements in contemporary care for neurotrauma patients.
